# Comparison of different airway pressures in (synchronized) ventilation during cardiopulmonary resuscitation in pigs

**DOI:** 10.1016/j.resplu.2026.101248

**Published:** 2026-01-29

**Authors:** Miriam Renz, Lea Müller, Jan Köhler, Roman Paul, Katja Mohnke, Andrea Urmann, Johanna Hain, René Rissel, Alexander Ziebart, Robert Ruemmler

**Affiliations:** a Department of Anesthesiology, University Medical Center of the Johannes Gutenberg University, Mainz, Germany; b Institute for Medical Biostatistics, Epidemiology and Informatics, University Medical Center of the Johannes Gutenberg University Mainz, Germany

**Keywords:** Cardiac arrest, Resuscitation, Ventilation, Synchronized ventilation, Porcine studies

## Abstract

**Background:**

The optimal ventilation strategy during cardiopulmonary resuscitation (CPR) remains undetermined. Synchronizing ventilation to chest compressions has been proposed to enhance end-organ perfusion and oxygenation. This study evaluates the pulmonary function and injury in a synchronized ventilation (SV) strategy using lower peak pressure compared to chest compression synchronized ventilation (CCSV) with higher peak pressures and intermittent positive pressure ventilation (IPPV).

**Materials/methods:**

35 pigs underwent cardiac arrest, followed by basic and advanced life support with mechanical chest compressions and ventilation according to randomized groups: IPPV (peak pressure (*P*_peak_) 40 mbar), SV (*P*_peak_ 20 mbar), and CCSV (*P*_peak_ 40 mbar). Arterial blood gases, ventilation-perfusion (V/Q) ratios, and hemodynamics were assessed during CPR and after return of spontaneous circulation (ROSC). Pulmonary histopathology and inflammatory markers (IL-6, TNF-α) were analyzed post-mortem.

**Results:**

During CPR, CCSV demonstrated superior oxygenation compared to SV (p_a_O_2_: CCSV 190.61 mmHg, SV 96.02 mmHg; *p* = 0.006), while CCSV and IPPV utilized significant higher airway pressures. SV showed the highest mean arterial pressure. Lactate levels were non-significantly highest in CCSV during CPR. ROSC rates were lower in CCSV (4/10) than in IPPV and SV (both 9/10); all non-ROSC CCSV cases exhibited pneumothoraces. Post-CPR, increased low V/Q and shunt fractions were observed in CCSV and SV exhibited reduced IL-6 expression.

**Conclusions:**

SV resulted in lower oxygenation but utilized lower airway pressures compared with CCSV and IPPV, produced less lactate, and achieved ROSC rates comparable to IPPV with fewer complications compared to CCSV. These findings suggest that SV may represent a viable alternative ventilation strategy during CPR. Further studies are needed to confirm these results.

## Introduction

In cardiac arrest, chest compressions and ventilatory support are widely accepted as essential measures for achieving a return of spontaneous circulation (ROSC).^1^ While mechanical ventilation is a thoroughly studied and well-established area in both critical care and perioperative medicine,^2^, ^3^, ^4^, ^5^ the optimal ventilation approach during cardiopulmonary resuscitation (CPR) remains an active area of research.^6^ Current guidelines from the European Resuscitation Council (ERC) and the American Heart Association (AHA) suggest a stepwise approach in airway management.^7^, ^8^ Once an advanced airway is placed both recommend to perform continuous chest compression with 10 breath per minute.^7^, ^8^ In the ERC guidelines mechanical ventilator settings are listed, such as a tidal volume of 6–8 mL kg^−1^ or a tidal volume to achieve a visible chest movement, a respiratory rate of 10 min^−1^, a positive end expiratory pressure (PEEP) 0–5 cm H_2_O and a peak pressure alarm at 60–70 cm H_2_O.^8^ Nevertheless, the authors of the 2025 ERC guidelines also note that the optimal method of ventilation during mechanical chest compressions remains uncertain.^8^

The primary goal of CPR is to maintain perfusion to vital organs to ensure adequate oxygen delivery. However, the interactions between changing thoracic pressures from chest compressions and ventilation can impact physiological parameters, including venous return, cardiac output and pulmonary function.^9^, ^10^, ^11^ Multiple approaches have been investigated, such as the use of automated ventilators and different ventilation modes.^6^ Among these, strategies such as ultra-low tidal volume ventilation,^12^ bi-level ventilation,^13^, ^14^ continuous positive airway pressure^15^, ^16^, ^17^ and others have been explored.^4^, ^6^ A novel technique, which synchronizes chest compressions to ventilation (CCSV) demonstrated potential benefits in enhancing blood flow and oxygenation compared to traditional ventilation techniques.^9^ Studies have highlighted both the positive effects of synchronization as well as the associated risks, such as pneumothoraces, which may be influenced by relatively high ventilatory pressures.^18^

In a previous porcine trial,^19^ we investigated the feasibility of a chest compression synchronized ventilation (CCSV) mode with peak pressures limited to 40 mbar and a customized experimental synchronized ventilation (SV) mode limited to 20 mbar. We demonstrated the feasibility of SV during CPR using significantly reduced inspiratory peak pressures, maintaining adequate oxygenation.^19^ However, low ROSC rates limited the evaluation of post-ROSC outcomes such as gas exchange, ventilation-perfusion (V/Q) ratios, and lung injury, highlighting the need for further investigation. The presented follow-up trial was conducted in two (CC)SV groups at different peak pressure levels and were compared to an intermittent positive pressure ventilation (IPPV). The primary endpoint was the pulmonary function, assessed via gas-exchange parameters and ventilation/perfusion (V/Q) ratios. Secondary endpoints included the assessment of pulmonary injury by performing histopathological analyses of lung tissue and quantifying pulmonary inflammation markers. The trial aims to provide further insights into the field to refine ventilation approaches during CPR.

## Materials and methods

### Preparations

This prospective randomized trial was approved by the State and Institutional Animal Care Committee Rhineland Palatine, Germany (approval no. G20-1-065). 35 landrace pigs (12–16 weeks, 28–34 kg) were examined. This trial compared two synchronized ventilation strategies with different peak pressures (CCSV and SV) to an asynchronous ventilation strategy (IPPV). The primary endpoint was pulmonary function, while secondary endpoints assessed lung injury and inflammation.

Sedation, transportation and preparation were carried out as previously detailed.^12^ In brief, general anesthesia was induced using intravenous injections of fentanyl, propofol and atracurium, followed by endotracheal intubation and ventilation with an intensive care respirator (Engstroem care station, GE healthcare, Munich). Intravascular sheaths were placed in the femoral arteries and veins bilaterally using ultrasound to establish invasive hemodynamic monitoring. Normothermia was obtained using body surface warming blankets.

For V/Q ratio measurements via Multiple Inert Gas Elimination Technique (MIGET), six chemically inert gases (sulfur hexafluoride, krypton, desflurane, enflurane, diethyl ether, acetone), characterized by distinct transpulmonary elimination constants, were dissolved in saline at nontoxic concentrations and administered intravenously for 30 min to achieve steady-state conditions. Afterwards, the animals were administered muscle relaxants and a fibrillation catheter was transvenously placed into the right atrium. Immediately prior to the intervention, animals were randomized into IPPV (*n* = 10), CCSV (*n* = 10), SV (*n* = 10) or sham (*n* = 5).

### Intervention

**Fig. 1:**
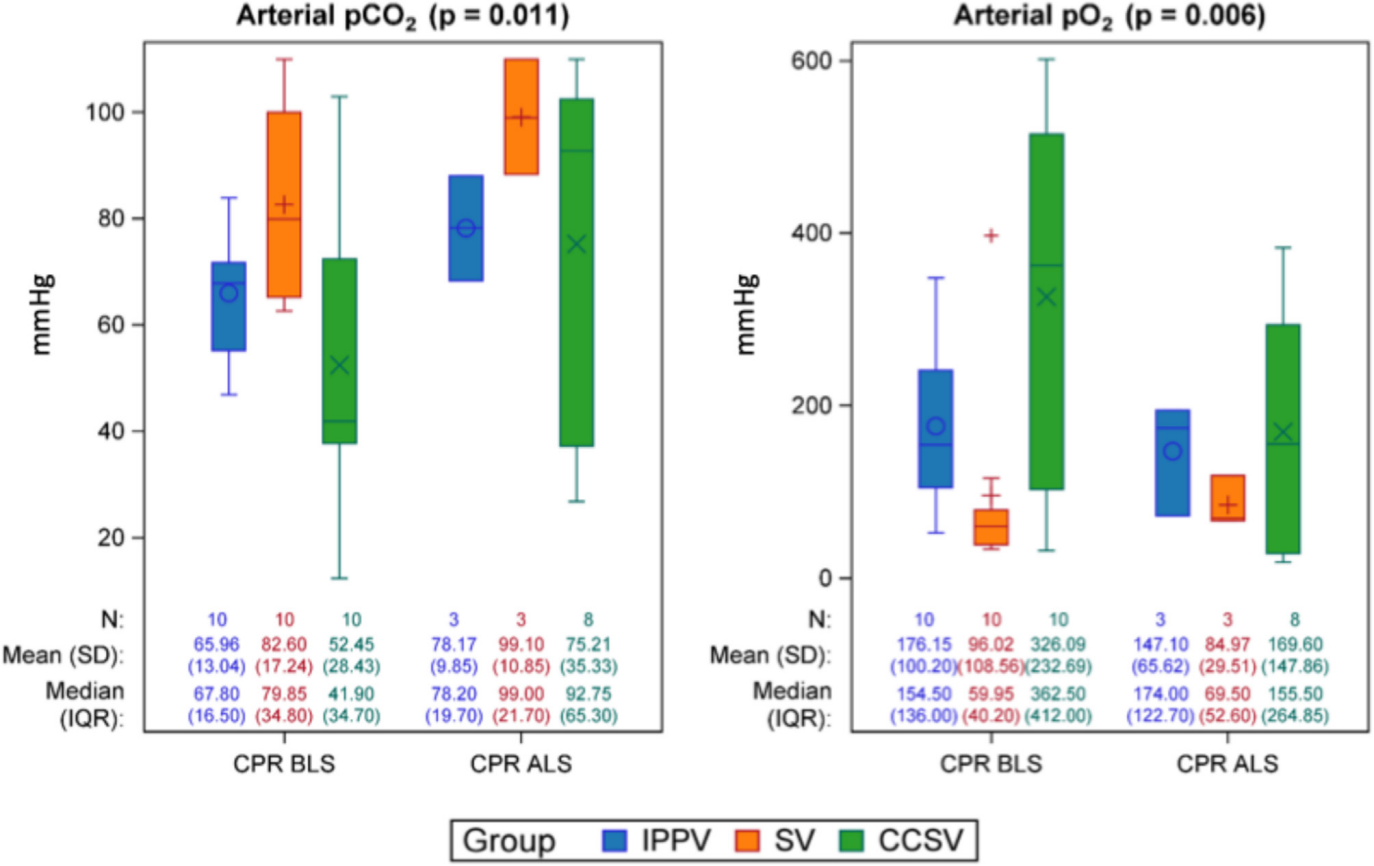
**Arterial blood gases**. During intervention, partial pressure of carbon dioxide (p_a_CO_2_) was significantly highest in SV (*p* = 0.011). Partial pressure of oxygen (p_a_O_2_) showed significant highest values in CCSV (*p* = 0.006) during CPR. Post-ROSC, there was no significant differences between the groups (data not shown in the graph). *N*: Number of observations at each time point. *P*-values are based on Type-III-tests of the fixed group effects of a mixed model. Partial pressures in mmHg. Values are presented as Mean (standard deviation). IPPV (intermittent positive pressure ventilation), SV (synchronized ventilation), CCSV (chest compression synchronized ventilation), sham (control group). BLS (basic life support), ALS (advanced life support), CPR (cardiopulmonary resuscitation), T0–T6 (measurement timepoints in hours after return of spontaneous circulation).

Ventricular fibrillation (VF) was induced using the fibrillation catheter and a flicker frequency between 50 and 200 Hz. After successful induction of VF, a no-flow time of 2 min was allowed. Following this period, basic life support (BLS) was conducted by performing mechanical chest compressions using the LUCAS-2-System (Stryker, Kalamazoo, MI, USA) with a frequency of 100/min. The interventional ventilation was performed according to the randomized intervention group (Table 1) using a Medumat ventilator (type MEDUMAT Standard^2^, Weinmann Emergency Medical Technology GmbH + Co. KG, Germany). After 5 min of BLS, blood gas analyses (radiometer, ABL90flex, Copenhagen, Denmark) and MIGET measurements (MMIMS-MIGET, Oscillogy LLC, Philadelphia, PA, USA) were performed, then advanced life support (ALS) was initiated. ALS was performed according to current guidelines, with minor adaptations. Following the rhythm analysis, persisting VF resulted in defibrillation (200 J bi-phasic, Zoll M-Series) followed by a 2 min-compression cycle. Epinephrine (1 mg) and vasopressine (0.1 U/kg) were administered each cycle with a bolus of amiodarone (150 mg) after the third defibrillation. Arterial blood gas samples were obtained after the third and sixth rhythm analyses. Ventilation was continued during ALS exactly as in BLS – according to the assigned randomized ventilation group (Fig. 1 supplement).

**Table 1:** Intervention groups with *n* = 10 animals per group. Abbreviations: IPPV (intermittent positive pressure ventilation), SV (synchronized ventilation), CCSV (chest compression synchronized ventilation).

**Group**	**IPPV (*n* = 10)**	**SV (*n* = 10)**	**CCSV (*n* = 10)**
Peak pressure (*P*_peak_)	40 mbar	20 mbar	40 mbar
Respiratory rate	10 breaths/min	chest compression rate	chest compression rate
Fraction of inspired oxygen (F_i_O_2_)	1.0	1.0	1.0
Trigger (TriggerInsp.)	5	5	5
Positive endexpiratory pressure (PEEP)	5 mbar	3 mbar	3 mbar

Hemodynamic (Datex Ohmeda S5 monitor, GE Healthcare, Munich, Germany) data were continuously recorded. Ventilatory data were also continuously recorded and extracted from the Medumat ventilator.

### Follow-up (post-ROSC)

If no ROSC was achieved after the sixth defibrillation, the trial was terminated. If ROSC was achieved, the ventilation was transitioned back from Medumat to the intensive care respirator, with a respiratory rate adjusted to maintain end-tidal carbon dioxide levels below 45 mmHg. Tidal volume (*V_t_*) was set to 6–8 ml/kgBW. Catecholamine administration was guided by a target mean arterial pressure (MAP) above 60 mmHg, with norepinephrine titrated as needed. The continuous recording of ventilation data and hemodynamics was maintained. Blood gas samples were collected hourly, and MIGET measurements were taken 10 min after ROSC, as well as 2 h and 6 h post-ROSC. The post-ROSC monitoring period lasted for 6 h, during which the animals were kept under general anesthesia throughout. The trial was terminated by euthanizing the animals using high doses of propofol and potassium chloride.

### Post-mortem

Lung tissue samples were collected from the upper and lower right lobes, both ventrally and dorsally. Samples were fixed in 4% formalin, paraffin-embedded, sectioned (2 μm), and HE-stained by the Tissue Bank of the University Medical Center Mainz. Histological evaluation was performed using an Olympus microscope (CX43RF, Olympus Cooperation, Tokyo Japan) with a CellSens Software (CellSens Entry.lnk, creation date 03.12.2018) and evaluated using the diffuse alveolar damage (DAD) score.^20^, ^21^ This score differentiates between seven characteristics: overdistension, epithelial destruction, inflammatory infiltration, alveolar edema, hemorrhage, interstitial edema and microatelectasis. The assessment of the DAD score was performed in an investigator-blinded manner. Additional lung tissue samples were snap-frozen for biomolecular analysis. RNA extraction and RT-PCR, using cyclophilin A (peptidyl-prolyl isomerase A, PPIA) as a reference gene, were conducted to quantify interleukin 6 (IL-6) and tumor necrosis factor alpha (TNFα) mRNA-expression with a LightCycler 480 system (Roche, Mannheim, Germany) per manufacturer’s protocol.

Animals in the control group (sham, *n* = 5) were monitored under general anesthesia for 6 h after the aforementioned baseline preparations, following the same experimental protocol without resuscitation.

### Statistics

The statistical analysis was conducted using SAS version 9.4 (SAS Institute, Cary, NC). For continuous data, the mean and standard deviation were reported, as well as descriptive box plots. Differences in ROSC were assessed using Fisher’s exact test. Differences in intervention groups separated by each outcome parameter were assessed using linear mixed models for repeated measurements. Fixed effects included in these models were type of intervention, time point of measurement and the interaction of intervention and time point. For post-mortem parameters, the anatomical regions (lobe, ventral/dorsal) of sample extraction were included as fixed effects. The individual animals were included as random effects in all models to account for the repeated measurements and therefore obtain correct standard errors. The intervention period (BLS, ALS) and follow-up period were modeled separately, as well as post-mortem histopathological damage parameters and biomarkers. The differences of the models’ overall least squared means were used to estimate pairwise group differences. Statistical significance was defined as a *p*-value of less than 0.05. Since no adjustment for multiple testing was performed, *p*-values are considered exploratory. Group differences are presented as “Estimate, [Lower/Upper 95%-confidence limit], *p*-value”. Descriptive values are presented as “value (standard deviation)”.

## Results

### Intervention

#### Gas exchange parameters

**Fig. 2:**
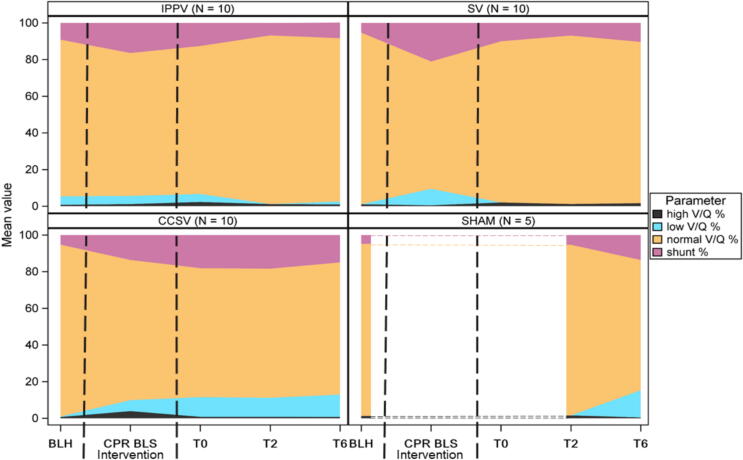
**Ventilation/perfusion measurements**. During intervention, ventilation/perfusion (V/Q) analysis showed most high V/Q in CCSV. No significant differences were observed in low or normal V/Q or in shunt fraction during CPR. During follow-up, CCSV showed highest fractions of low V/Q and shunt, as well as a lower fractions of normal and high V/Q compared to the other intervention groups. Ventilation/Perfusion (V/Q) values in % (*y*-axis). IPPV (intermittent positive pressure ventilation), SV (synchronized ventilation), CCSV (chest compression synchronized ventilation), sham (control group). BLH (Baseline), BLS (basic life support), CPR (cardiopulmonary resuscitation), T0, T2 and T6 (measurement timepoints in hours after return of spontaneous circulation).

All groups showed impaired gas exchange during CPR (Fig. 1). In CCSV, p_a_CO_2_ was significantly lower and p_a_O_2_ was significantly higher compared to SV and non-significantly to IPPV (Fig. 1). The V/Q analyses revealed a significantly greater proportion of high V/Q in CCSV compared to SV (3.45 [1.59; 5.31]%, *p* < 0.001) and IPPV (2.80 [0.94; 4.66]%, *p* = 0.005) (Fig. 2). No significant differences were observed in low or normal V/Q or in shunt fraction (Fig. 2).

#### Ventilatory parameters

**Fig. 3:**
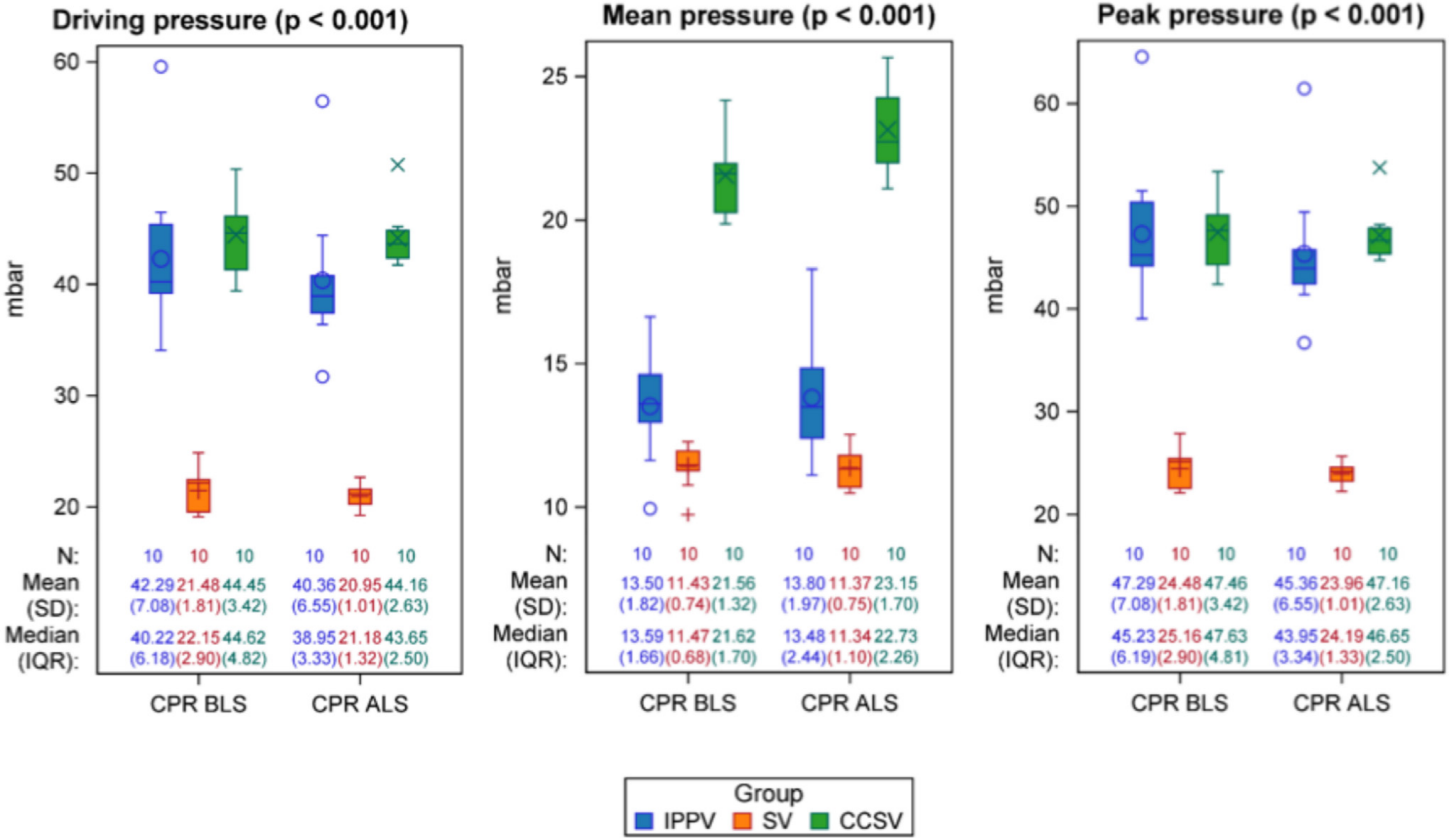
**Airway pressures**. During CPR, intervention groups with high peak pressure (IPPV, CCSV) showed significantly higher peak and driving pressures compared to SV (*p* < 0.001). Additionally, mean airway pressure was significantly highest in the CCSV (*p* < 0.001) during CPR. *N*: Number of observations at each time point. *P*-values are based on Type-III-tests of the fixed group effects of a mixed model. Pressures in mbar. IPPV (intermittent positive pressure ventilation), SV (synchronized ventilation), CCSV (chest compression synchronized ventilation), sham (control group). BLS (basic life support), ALS (advanced life support), CPR (cardiopulmonary resuscitation), T0 to T6 (measurement timepoints in hours after return of spontaneous circulation).

Regarding airway pressures, both high-pressure intervention groups – IPPV and CCSV – exhibited significantly elevated peak pressures compared with SV (IPPV vs. SV: 22.11 [18.46; 25.76] mbar, *p* < 0.001, CCSV vs. SV: 23.09 [19.45; 26.74] mbar, *p* < 0.001) as well as driving pressures (IPPV vs. SV: 20.11 [16.46; 23.76] mbar, *p* < 0.001, CCSV vs. SV: 23.09 [19.45; 26.74] mbar, *p* < 0.001) during CPR (Fig. 3). Additionally, mean airway pressure was significantly highest in CCSV (vs. IPPV: 8.70 [7.58; 9.82] mbar, *p* < 0.001; vs. SV 10.95 [9.84; 12.07] mbar, *p* < 0.001) (Fig. 3). Respiratory rates in the synchronized ventilation groups should match the frequency of mechanical chest compressions. However, different respiratory rates were observed between CCSV (descriptive data: BLS: 82.88 (15.12)/min, ALS: 82.61 (18.58)/min) and SV (descriptive data: BLS: 30.75 (35.54)/min, ALS: 30.60 (30.85)/min)). CCSV showed significantly higher respiratory rates than IPPV (72.30 [56.17; 88.44]/min, *p* < 0.001) and SV (51.24 [35.40; 67.08]/min, *p* < 0.001). Tidal volumes were significantly increased in IPPV compared to CCSV (82.31 [45.30, 119.33] ml, *p* < 0.001) and SV (139.72 [102.61; 176.84] ml, *p* < 0.001). SV showed also significantly lower tidal volumes than CCSV (57.41 [20.29; 94.53] ml, *p* = 0.004). Minute volumes were highest in CCSV compared to IPPV (5.80 [4.07; 7.52] L/min, *p* < 0.001) and SV (6.32 [4.58; 8.05] L/min, *p* < 0.001).

#### Circulatory parameters

**Fig. 4:**
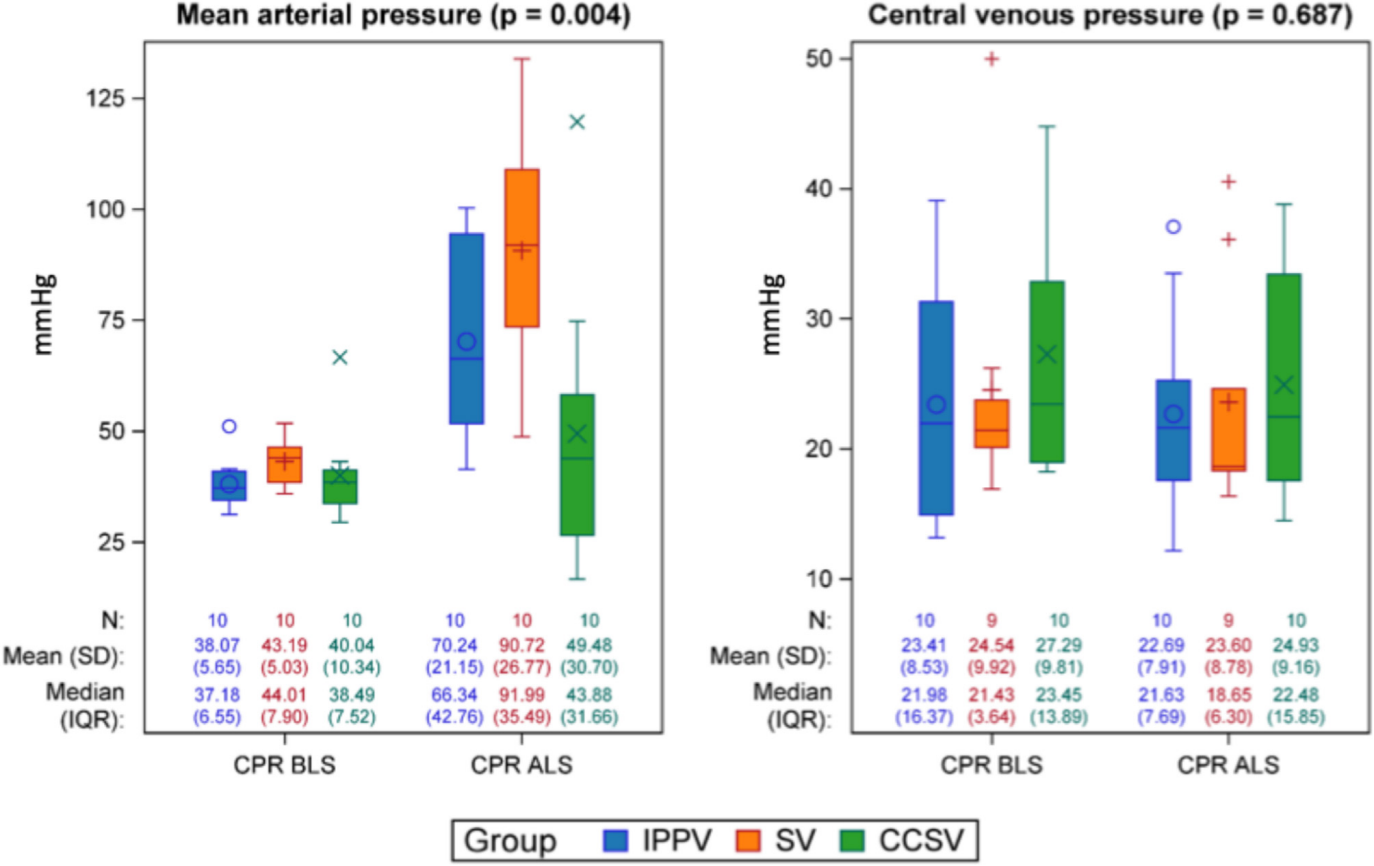
**Hemodynamics**. During intervention, the mean arterial pressure was significantly highest in SV (*p* = 0.004). The central venous pressure showed no significant differences between the intervention groups or the sham group. *N*: Number of observations at each time point. *P*-values are based on Type-III-tests of the fixed group effects of a mixed model. Hemodynamic values in mmHg. IPPV (intermittent positive pressure ventilation), SV (synchronized ventilation), CCSV (chest compression synchronized ventilation), sham (control group). BLS (basic life support), ALS (advanced life support), CPR (cardiopulmonary resuscitation), T0–T6 (measurement timepoints in hours after return of spontaneous circulation).

Hemodynamic parameters varied across groups. During BLS, the mean arterial pressure (MAP) decreased in all three groups and stayed low in CCSV during ALS. Irrespective of timepoint CCSV showed a MAP of 44.75 (20.51) mmHg, while IPPV showed a MAP of 54.16 (13.40) mmHg (CCSV vs. IPPV: 9.40 [−3.08; 21.88]/mmHg, *p* = 0.134). SV showed a MAP of 66.96 (15.90) mmHg irrespective of timepoint (CCSV vs. SV: 22.20 [9.72; 34.68;]/mmHg, *p* = 0.001; IPPV vs. SV: 12.80 [0.32; 25.28]/mmHg, *p* = 0.045)) (Fig. 4). The central venous pressure was highest in CCSV without reaching statistical significance (Fig. 4). Lactate levels increased in all groups during intervention and were highest in CCSV (descriptive data: BLS 5.40 (2.33) mmol/l, ALS 7.85 (2.50) mmol/l), displaying no significant differences (CCSV vs. IPPV: 0.44 [−1.22; 2.09], *p* = 0.591], CCSV vs. SV: 1.09 [−0.52; 2.71], *p* = 0.175, IPPV vs. SV: 0.66 [−1.00; 2.31], *p* = 0.421) between the groups (descriptive data: SV: BLS 4.02 (1.29) mmol/l, ALS 6.46 (1.57) mmol/l; IPPV: BLS 4.51 (1.91) mmol/l, ALS 6.96 (0.51) mmol/).

### Follow-up (post-ROSC)

Oxygenation values and P/F ratio (p_a_O_2_/FiO_2_) improved across all groups within 1–2 h post-ROSC, with sham exhibiting the highest values. CCSV showed the highest P/F ratio among the intervention groups compared to IPPV (91.32 [1.96; 180.68], *p* = 0.045) and SV (76.02 [13.34; 165.38], *p* = 0.093). Still, the P/F ratio in all groups was constantly above 300.

Post-ROSC, CCSV showed significantly higher proportions of low V/Q and shunt, as well as a lower fraction of normal and high V/Q compared to the other intervention groups (Fig. 2).

Lactate levels decreased in all groups after CPR. Around 4 h post-ROSC, all groups demonstrated lactate levels below 2 mmol/l.

Peak, mean and driving pressures were elevated in all intervention groups after ROSC, with no statistically significant differences between the groups (Peak: CCSV vs. IPPV: −2.67 [−6.40; 1.05], *p* = 0.151; CCSV vs. SV: −2.20 [−5.92; 1.52], *p* = 0.234; IPPV vs. SV: 0.47 [−2.45; 3.39], *p* = 0.740. Mean: CCSV vs. IPPV: −1.00 [−2.44; 0.43], *p* = 0.161; CCSV vs. SV: −0.84 [−2.28; 0.59], *p* = 0.237; IPPV vs. SV: 0.16 [−0.96; 1.29], *p* = 0.769. Driving: CCSV vs. IPPV: −1.84 [−5.47; 1.78], *p* = 0.303; CCSV vs. SV: −0.67 [−4.29; 2.95], *p* = 0.706; IPPV vs. SV: 1.18 [−1.66; 4.02], *p* = 0.400). However, most of the time airway pressures were significantly higher in the intervention groups versus sham (Peak: CCSV vs. SHAM: 3.75 [−0.40; 7.91], *p* = 0.075; IPPV vs. SHAM: 6.43 [2.97; 9.88], *p* < 0.001; SV vs. SHAM: 5.95 [2.50; 9.41], *p* = 0.002. Mean: CCSV vs. SHAM: 1.25 [−0.35; 2.85], *p* = 0.120; IPPV vs. SHAM: 2.25 [0.92; 3.58], *p* = 0.002; SV vs. SHAM: 2.09 [0.76; 3.42], *p* = 0.004. Driving: CCSV vs. SHAM: 4.38 [0.34; 8.42], *p* = 0.035; IPPV vs. SHAM: 6.23 [2.87; 9.59], *p* < 0.00; SV vs. SHAM: 5.05 [1.69; 8.41], *p* = 0.005) (Fig. 3).

#### Postmortem

In IPPV and SV, 9 animals reached ROSC, in contrast to 4 animals in CCSV (90% IPPV, 90% SV, 40% CCSV; *p* = 0.022). All non-ROSC animals in CCSV had unilateral pneumothoraces; one case also occurred in IPPV.

**Fig. 5:**
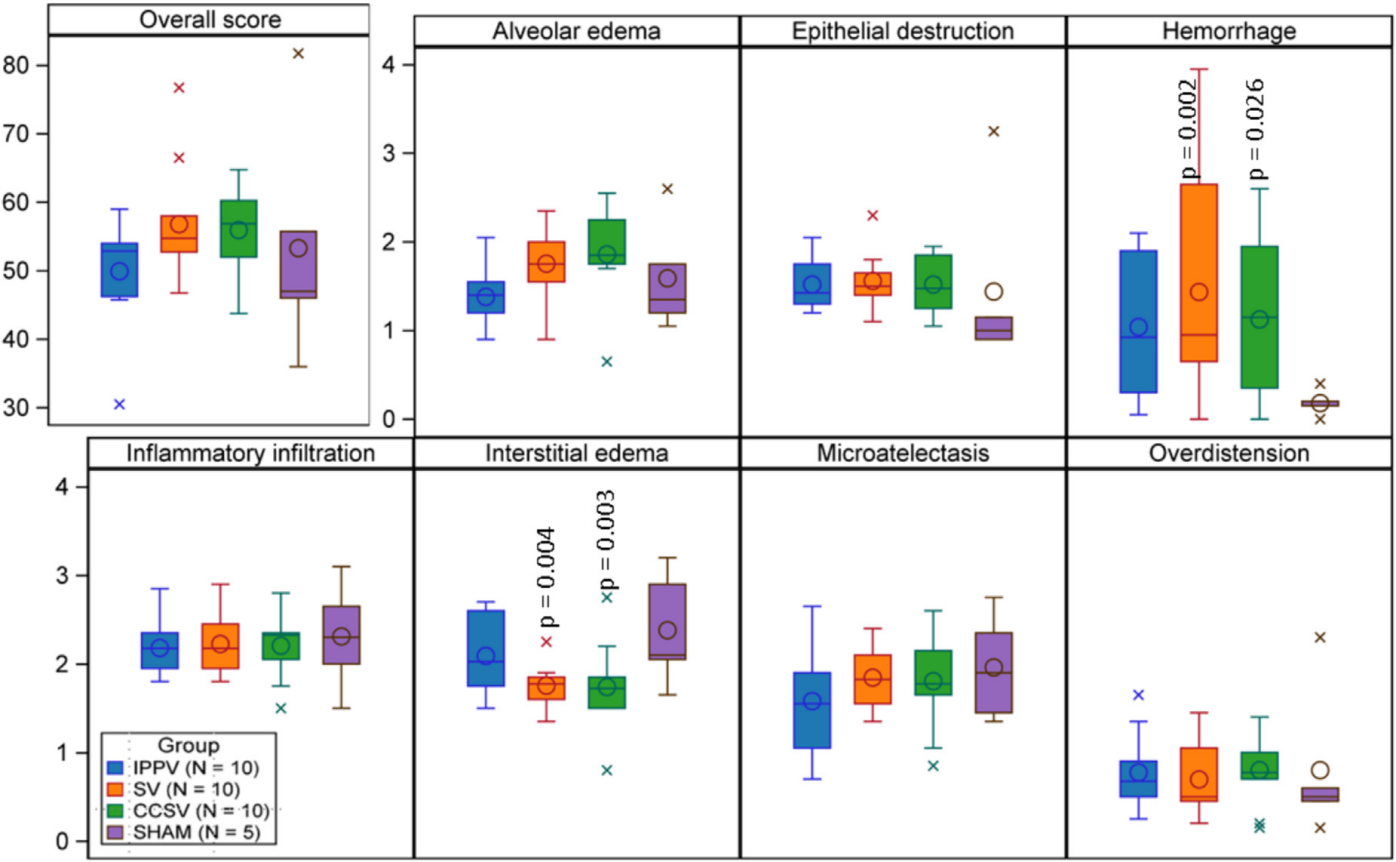
**Boxplots displaying diffuse alveolar damage (DAD) scoring**. The DAD-score was evaluated postmortem. It differentiates between seven characteristics: alveolar edema, epithelial destruction, hemorrhage, inflammatory infiltration, interstitial edema, microatelectasis and overdistension. *P*-values based on Wald-test performed on mixed model for repeated measurements.

Histopathological damage was evaluated using the DAD score. Regardless of the lung region, more hemorrhage (SV vs. sham *p* = 0.013, IPPV vs. sham *p* = 0.001, CCSV vs. sham *p* = 0.026) and slightly more epithelial destruction was seen in all three intervention groups when compared to sham. No significant differences were seen in other categories such as overdistension or microatelectasis (Fig. 5).

The evaluation of pulmonary inflammation, using mRNA expression of IL-6 and TNFα, showed significantly higher values of IL-6 in CCSV (CCSV vs. IPPV: 0.00014 [0.00003; 0.00025], *p* = 0.006; CCSV vs. SV: −0.00016 [−0.00026; −0.00005], *p* = 0.002).

## Discussion

In this animal trial, we investigated a ventilation approach designed to synchronize ventilation with chest compressions using low ventilation pressure (SV) and compared it to intermittent positive pressure ventilation (IPPV) and a previously established synchronized approach with higher peak pressure (CCSV).

SV maintained sufficient oxygenation, lower lactate, improved hemodynamics and high ROSC rates with minimal complications. CCSV had optimized gas exchange while displaying higher airway pressures and lower ROSC with more complications. IPPV showed intermediate airway pressures and tidal volumes, also high ROSC rates and low pulmonary inflammation.

Synchronizing ventilation with chest compressions has been suggested to benefit cardiac and thoracic pump mechanisms.^11^ These concepts propose that blood flow during CPR results either from direct ventricular compression or from increased intrathoracic pressure during compression.^11^, ^22^ Synchronization is expected to increase intrathoracic volume, thereby supporting both the cardiac and thoracic pump mechanisms and potentially enhancing cardiac output and oxygenation.^9^, ^23^ However, high respiratory rates combined with low tidal volumes may raise concerns about dead space and insufficient alveolar ventilation. In this trial, the CCSV mode yielded a more effective gas exchange during CPR compared to SV, with significantly optimized decarboxylation and oxygenation. In contrast, SV maintained p_a_O_2_ levels close to 100 mmHg, which are generally sufficient to ensure adequate tissue oxygenation. In general, oxygen supply is essential during CPR to support mitochondrial respiration and improve the likelihood of ROSC as well as improved survival rates to hospital admission, but the optimal arterial oxygen level remains unclear.^24^ In theory, high inspired oxygen levels can enhance oxygen delivery, but excessive oxygen levels may increase reactive oxygen species and could potentially contribute to tissue injury.^24^ The asynchronous ventilation strategy IPPV led to sufficient gas exchange during CPR but did not achieve the same quality of gas exchange as observed with CCSV. The enhanced oxygenation observed with CCSV can be attributed to the elevated mean airway pressures inherent to this ventilation strategy; however, these same pressures likely promoted barotrauma, reflected by the higher pneumothorax incidence and lower ROSC rate. A similar finding of improved oxygenation, but with a greater occurrence of pneumothoraces, was also observed during prolonged experimental cardiopulmonary resuscitation in a porcine model when ventilation was synchronized with chest compressions.^18^ These findings highlight the delicate balance between improved oxygenation and an increased risk of barotrauma. Mechanical ventilation (during CPR) can cause pulmonary injuries if airway pressures or tidal volumes are too high.^25^, ^26^ Excessive pressures can lead to barotrauma and increase the risk of pneumothorax and may impair ROSC, highlighting the need for careful pressure control. In the high-pressure synchronized ventilation group (CCSV), the ROSC-rate was significantly lower than in SV and IPPV. All non-ROSC animals in CCSV had pneumothoraces, with one case of tension pneumothorax likely preventing adequate circulation. Similarly, another study investigating chest compression-synchronized ventilation during prolonged CPR reported a significant incidence of severe pneumothoraces.^18^ The high pressure group IPPV showed a higher ROSC-Rate compared to CCSV. This observation could be explained by the fact that not only the high peak airway pressure alone leads to barotrauma, but that the high respiratory rate and increased mean airway pressure in CCSV further exacerbate the trauma, resulting in complications such as pneumothorax occurring more frequently in CCSV compared to IPPV. SV also showed a higher ROSC-Rate then CCSV, which could be explained by the lower airway pressures and the reduced risk of barotrauma.

Volutrauma can be caused by excessive tidal volumes leading to alveolar overdistension.^2^, ^26^ Compared with CCSV, SV generated lower tidal volumes likely due to pressure limitation and was associated with lower IL-6 levels, suggesting reduced pulmonary inflammation.^25^ IPPV showed similarly low IL-6 levels. Lung protective ventilation is aiming to put as little strain and stress as possible to the lungs to reduce VILI. Mechanical power (MP) is a concept that encompasses all VILI parameters, such as elastic, dynamic and resistive components.^27^ However, MP is expressed in joules/minute, meaning that all aforementioned components directly depend on the respiratory rate.^27^, ^28^ This could suggest that any potential benefits caused by lower tidal volumes might eventually be set off by higher total energy deposited in lung tissue.

Effects of MP and pulmonary trauma and inflammation can affect alveolar ventilation and perfusion. However, the matching of perfusion and ventilation to ensure effective gas exchange is important. To assess this, V/Q ratios were analyzed using the MIGET. It measures V/Q ratios by analyzing the differential alveolar elimination of infused inert gases at varying solubilities.^29^, ^30^, ^31^ The method has previously been established during CPR and allows to determine precise values for high, normal and low V/Q ratios and shunt fraction.^30^ During CPR, CCSV exhibited a significantly higher proportion of high V/Q, suggesting increased pulmonary inflation and possible overdistension, potentially due to higher minute volumes. High V/Q ratios, reflecting overinflated lung regions, could alter intrathoracic pressures and thus modulate blood flow during chest compressions, linking pulmonary mechanics to systemic perfusion outcomes. Within the heart and thoracic pump concepts, blood flow is impacted not only by chest compressions and decompressions but also by ventilation.^22^ The pressures generated by ventilation, along with their interaction with thoracic compressions, play a role in modulating blood flow.^22^, ^32^ In our trial, MAP initially decreased in all groups during CPR. However, CCSV showed a more impaired MAP throughout ongoing CPR, while SV and IPPV displayed an increasing MAP while ongoing CPR. IPPV exhibited V/Q ratios similar to SV and a slightly lower MAP, yet still maintained more optimal MAP than CCSV. Altered perfusion during CPR can lead to oxygen deprivation in end organs, resulting in increased blood lactate levels.^33^ While high lactate levels during CPR are associated with poor one-month survival, the exact threshold remains undefined, and its prognostic value is still debated.^33^, ^34^, ^35^ In the presented trial, lactate levels increased in all intervention groups during CPR as expected, but the rise was less pronounced in SV. Taken together, these findings suggest that the application of lower ventilation pressures in synchronized ventilation like in SV does not adversely affect perfusion during CPR, whereas CCSV was associated with higher mean airway pressures, more high V/Q ratios, impaired MAP, and elevated lactate levels during CPR. In asynchronous ventilation (IPPV) V/Q ratios were similar to SV and a slightly lower but still optimal MAP was seen, with correspondingly less lactate like in SV during CPR.

Post-ROSC, CCSV showed the lowest high and normal V/Q fractions, as well as increased low V/Q and shunt fractions, suggesting that previously overdistended regions impaired pulmonary function post-ROSC and may be collapsed into atelectasis. Yet, these findings have to be interpreted with caution, as only 40% in CCSV achieved ROSC. Additionally, the absolute amount of potentially overdistended lung tissue in CCSV is still very low, which might render it clinically irrelevant. Although lactate levels returned to normal within the first 4 h post-ROSC in all groups, CCSV showed a non-significantly slower decrease than SV. This could be explained by an increased cardiac output immediately after CPR in SV, potentially leading to improved organ perfusion. Another marker for oxygenation efficiency is the P/F ratio, which was assessed also post CPR. Although the P/F ratio was reduced in all intervention groups post-ROSC compared to sham, all intervention groups maintained values within physiological range, indicating sufficient oxygenation post-ROSC.

## Limitations

This trial has several limitations. The number of animals per group was increased to *n* = 10 compared to the previously described pilot trial (*n* = 5) to enhance the robustness and reproducibility of findings, given the biological variability observed in vivo. Although a formal power analysis was not conducted, doubling the sample size was intended to reduce the likelihood of Type II-errors and to ensure that moderate effects could be reliably detected. This approach aligns with current best practices in experimental design for large animal trials, aiming to balance statistical confidence with ethical considerations. Another limitation observed was, that in the synchronized groups, given no technical issues, the respiratory rates should have been identical. However, possibly due to the low-pressure limitation, some breaths in SV were not delivered, conclusively leading to a lower respiratory rate maybe due to insufficient triggering of the ventilator. Also limiting are is missing data, which occurred at some measurement timepoints during CPR because animals reached ROSC at different timepoints during the intervention.

## Conclusion

Synchronized ventilation with lower airway pressures (SV) appears to be a viable alternative ventilation strategy to asynchronous IPPV or synchronized ventilation using higher airway pressures (CCSV) during CPR. SV resulted in lower oxygenation during CPR, however lactate increased less in SV than with CCSV, indicating preserved pulmonary function and not negatively impacted organ perfusion. SV and IPPV demonstrated a high ROSC rate and fewer complications such as pneumothoraces and inflammation compared to CCSV. Overall, these findings support SV as a promising alternative ventilation strategy during CPR, although further studies are needed to needed to confirm these results.

## CRediT authorship contribution statement

**Miriam Renz:** Writing – original draft, Resources, Methodology, Investigation, Formal analysis, Data curation, Conceptualization. **Lea Müller:** Investigation, Data curation. **Jan Köhler:** Investigation, Data curation. **Roman Paul:** Writing – review & editing, Visualization, Formal analysis, Data curation. **Katja Mohnke:** Investigation, Data curation. **Andrea Urmann:** Investigation, Data curation. **Johanna Hain:** Investigation, Data curation. **René Rissel:** Investigation, Data curation. **Alexander Ziebart:** Supervision, Resources, Project administration. **Robert Ruemmler:** Writing – review & editing, Supervision, Resources, Project administration, Funding acquisition, Data curation, Conceptualization.

## Declaration of competing interest

The authors declare that they have no known competing financial interests or personal relationships that could have appeared to influence the work reported in this paper.
