# Supplementary material for: Comparison of different airway pressures in (synchronized) ventilation during cardiopulmonary resuscitation in pigs
Source: Resusc Plus. 2026 Jan 29;28:101248. doi: 10.1016/j.resplu.2026.101248 (PMC12907900; doi:10.1016/j.resplu.2026.101248)
Supplement: Supplementary Fig. 1 [file mmc1.docx]

**Figure 1 supplement:**

Schematic overview of the experimental protocol. After preparations, ventricular fibrillation was induced followed by 2 minutes of no-flow time (*). Basic Life Support (BLS) was then performed for 5 minutes performing mechanical chest compressions at 100/minute and ventilation according to the randomized intervention group. Blood gas analyses and Multiple Inert Gas Elimination Technique (MIGET) measurements were conducted after 5 minutes of BLS. Advanced Life Support (ALS) followed, with a maximum of 6 defibrillations, continued mechanical chest compressions (100/minute), ventilation per assigned group, rhythm analysis every 2 minutes, and administration of epinephrine, vasopressin, and amiodarone as indicated. Arterial blood gases were sampled after the 3rd and 6th rhythm analyses. Animals were either monitored for 6 hours post-Return of Spontaneous Circulation (ROSC) or if ROSC was not reached directly underwent post-mortem analysis.

For the control group (sham), the animals underwent the preparatory procedures and were then directly transitioned to the post-ROSC monitoring period (dashed arrow).
